# Perceived Occupational Stress is associated with Decreased Cortical Activity of the Prefrontal Cortex: A Multichannel Near-infrared Spectroscopy Study

**DOI:** 10.1038/srep39089

**Published:** 2016-12-13

**Authors:** Po-Han Chou, Wei-Hao Lin, Chao-An Hung, Chiung-Chih Chang, Wan-Rung Li, Tsuo-Hung Lan, Min-Wei Huang

**Affiliations:** 1 Department of Psychiatry, Taichung Veterans General Hospital, Taichung, Taiwan; 2 Department of Psychiatry, Faculty of Medicine of Medicine, National Yang-Ming University, Taipei, Taiwan; 3 Department of Photonics, National Chiao Tung University, Hsinchu, Taiwan; 4 Department of Biological Science and Technology, National Chiao Tung University, Hsinchu, Taiwan; 5 Department of Nursing, Chung Shan Medical University Hospital, Taichung, Taiwan; 6 School of Nursing, Chung Shan Medical University, Taichung, Taiwan; 7 Department of Neurology, Cognition and Aging Center, Kaohsiung Chang Gung Memorial Hospital, Chang Gung University College of Medicine, Kaohsiung, Taiwan; 8 Chia-Yi branch, Taichung Veterans General Hospital, Chiayi City, Taiwan

## Abstract

Despite an increasing number of reports on the associations between chronic occupational stress and structural and functional changes of the brain, the underlying neural correlates of perceived occupational stress is still not clear. Perceived stress reflects the extents to which situations are appraised as stressful at a given point in one’s life. Using near-infrared spectroscopy, we investigated the associations between perceived occupational stress and cortical activity over the bilateral frontotemporal regions during a verbal fluency test. Sixty-eight participants (17 men, 51 women), 20–62 years of age were recruited. Perceived occupational stress was measured using the Chinese version of Job Content Questionnaire, and the Chinese version of the Copenhagen Burnout Inventory. We found statistically significant negative associations between occupational burnout and brain cortical activity over the fronto-polar and dorsolateral prefrontal cortex during the VFT (r = −0.343 to −0.464). In conclusion, our research demonstrated a possible neural basis of perceived occupational stress that are distributed across the prefrontal cortex.

Recently, excessive workloads and job stress have become major public health concerns worldwide. Chronic exposure to workplace stress has been associated with adverse mental health problems such as depression^1^. The Job Demand-Control (JDC) model, proposed by Karasek^2^, is a well-established model for the relationship between job stress and health posing that excessive work demands and loss of control at work contribute to burnout. People with occupational burnout may complain of symptoms including sleeplessness, memory and concentration problems, diffuse aches, profound fatigue, irritability, and feeling emotionally drained, which they often attribute to occupational stress^3^. However, the underlying mechanism remains unknown.

Previous neuroimaging and neurobiology studies have consistently implicated the role of the prefrontal cortex (PFC) in stress regulation such as determining which events are experienced as threatening or potentially stressful, and stress adaptation^4^,^5^,^6^. For instance, using functional magnetic resonance imaging (fMRI), Pruessner *et al*.’s study implicated that the medial PFC in combination with the anterior cingulate cortex in the regulation of the human stress response during the Montreal Imaging Stress Task^7^. Qin and colleagues also reported that an experimentally induced acute stress among healthy individuals resulted in a reduction of working memory-related dorsolateral prefrontal cortex (DLPFC) activity^8^. However, few neuroimaging studies have investigated the associations between occupational stress and the brain.

In a positron emission tomography (PET) study, Ohira and colleagues revealed that participants with high chronic job stress exhibited diminished activity in the anterior caudate, orbitofrontal cortex, VLPFC, insula, and midbrain^9^. Furthermore, using near infrared spectroscopy (NIRS), Kawasaki and colleagues found a significant correlation between higher job demand and reduced cortical activity in the left DLPFC among female employees^10^. However, these studies did not examine self-reported occupational stress. Perceived stress reflects a global personal evaluation of stress experienced as a result of the stressful event and their subjective appraisal, inadequate coping resources, and a perceived lack of control^11^. Therefore, people may experience similar negative life events, but their appraisal of the associated psychological impact differs.

To the best of our knowledge, only a few neuroimaging studies have investigated the underlying neural correlates of subjective psychological reactions to occupational stress (i.e., occupational burnout). A structural MRI study showed that individuals with occupational burnout exhibited significantly reduced gray matter (GM) volumes of the anterior cingulate and DLPFC as well as reduced caudate and putamen volumes^12^. Moreover, Savic’s study found a significant thinning of the mesial frontal cortex and a more pronounced age-related thinning of the frontal cortex among individuals with occupational burnout^3^. In contrast, Rydmark *et al*. and Sandstrom *et al*. found no differences in prefrontal or hippocampal volumes between individuals with occupational burnout and controls^13^,^14^. With regard to fMRI studies, Golkar *et al* found that during acoustic startle reflex testing, the connectivity between amygdala and the DLPFC was significantly weaker among individuals with burnout^15^. Moreover, Sandstrom *et al*. found an under-recruitment of prefrontal cortical areas during cognitive tasks among female patients suffering from work-related long-term sick leave^16^. Durning and colleagues used two items of the Maslach Burnout Inventory, to measure emotional exhaustion and depersonalization. They found that during a clinical reasoning task, higher depersonalization scores were associated with diminished blood-oxygen-level dependent (BOLD) signal in the right DLPFC and middle frontal gyrus while reflecting on clinical problems and reduced BOLD signal in the bilateral precuneus while answering clinical problems in resident doctors^17^. Furthermore, in a PET study, Jovanovic and colleagues found that occupational stress was associated with a reduction of limbic 5-HT_1A_ receptor binding and a functional dysregulation of the ACC/mPFC^18^. However, due to heterogeneity in the sample compositions, assessments, and methodologies used in previous studies, the exact neural correlates of occupational burnout remains unclear.

Multi-channel NIRS is a recently developed functional neuroimaging technology that allows the non-invasive measurement of spatio-temporal neural activity. In comparison to existing imaging techniques, such as PET, SPECT, and fMRI, NIRS is easier to administer, tolerates small movements, inexpensive, and provides excellent time resolution and moderate spatial resolution^19^. More important, NIRS provides a bedside measurement of oxy-hemoglobin ([oxy-Hb]) and deoxy-hemoglobin ([deoxy-Hb]) concentrations, which are considered to indicate regional cerebral blood volumes and strongly correlate with fMRI signals^20^. Therefore, the aim of this study was to examine the relationship between perceived occupational stress and the prefrontal hemodynamic responses during a verbal fluency test (VFT). Based on the results of previous studies, we hypothesize that perceived occupational stress would be associated with reduced PFC activity during the VFT.

## Results

### Cortical activity during the VFT

**Figure 1:**
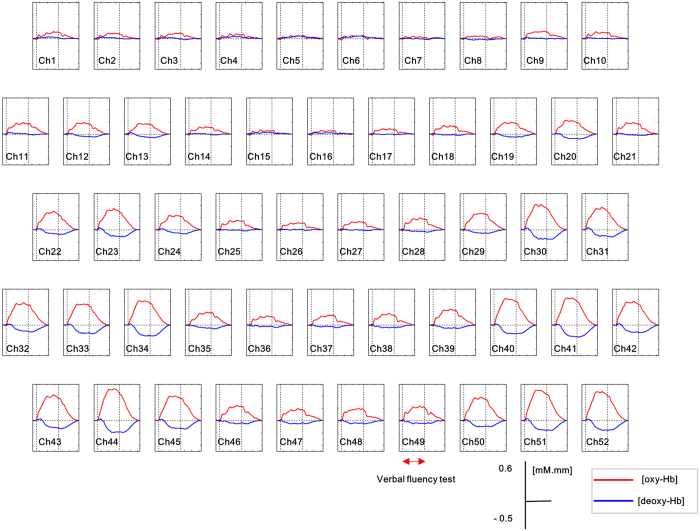
Grand average waveforms of hemoglobin concentration ([Hb]) changes during the verbal fluency task across all the subjects for 52 channels. [oxyHb] and [deoxyHb] are shown in red and blue, respectively.

There were significant increases in [oxy-Hb] changes during the cognitive task relative to the pre-task baseline over the bilateral frontotemporal regions at 51 channels (except ch7; FDR-corrected-*P* < 0.049; Fig. 1). In addition, significant decreases in [deoxy-Hb] changes were identified at 39 channels (ch1–2, ch4–6, ch12–13, ch15–16, ch19–20, ch22–24, and ch28–52; FDR-corrected-*P* < 0.038).

### Correlational analyses

**Figure 2:**
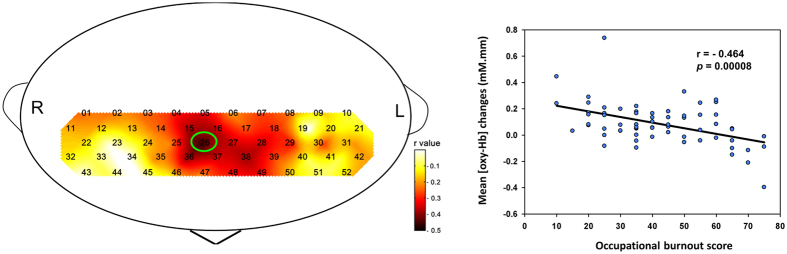
This figure illustrates the correlation coefficients (r) for 52 channels in study participants. The Arabic numerals represent channel locations. The scatter plot demonstrates the occupational burnout score regression slope for channel 26 (as denoted by a green circle), approximately located at the right fronto-polar region.

Nine channels demonstrated significant correlations between occupational burnout and reduced cortical activity (ch5, ch15–16, ch26–28, and ch36–38; FDR-corrected *P* < 0.0096; *r* = −0.343 to −0.464). These channels are located approximately at the bilateral fronto-polar cortex (FPC) and the DLPFC (Fig. 2). Among the parameters in the Chinese version of Job Content Questionnaire (C-JCQ), we did not find any relationship between cortical activity and job control. On the other hand, we found significantly negative correlation between cortical activity and job demand at ch16 (FDR corrected *P* < 0.00096; *r* = −0.412), and marginally significant correlations at ch26 (uncorrected *P* = 0.009; *r* = −0.316) and ch27 (uncorrected *P* = 0.012; *r* = −0.311). Ch16, 26, and 27 formed a cluster of channels located approximately at the fronto-polar region. Furthermore, no associations between job strain and mean [oxy-Hb] changes during the VFT were found.

Among those nine channels significantly correlated with occupational burnout, multiple regression analyses revealed significant contributions of occupational burnout in six channels (*β* = −0.311 to −0.426; uncorrected *P* = 0.000 to 0.010) among other variables (Table 1). In addition, for gender, significant contributions were found in four channels (*β* = 0.225 to 0.356; uncorrected *P* = 0.002 to 0.044), BDI-II scores in two channels (*β* = −0.430 to −0.457; both uncorrected *P* < 0.001), personal burnout scores in one channel (*β* = −0.318; uncorrected *P* = 0.006), and job demand in one channel (*β* = −0.412; uncorrected *P* < 0.001). Furthermore, SES, age, and VFT performance did not show significant associations with cortical activity.

### Correlational analyses of other clinical measurements

We found that occupational burnout was significantly associated with personal burnout (*r* = 0.633, *P* < 0.001), job strain (*r* = 0.374, *P* = 0.032), job demand (*r* = 0.515, *P* = 0.000) and BDI scores (*r* = 0.542, *P* < 0.001). Personal burnout was significantly positively correlated with BDI scores (*r* = 0.524, *P* < 0.001) and negatively correlated with VFT performance (*r* = −0.386, *P* = 0.001). Job demand (*r* = 0.345, *P* = 0.004) and job strain (*r* = 0.252, *P* = 0.038) were significantly associated with BDI scores, respectively. A significant association between mean [deoxy-Hb] changes and occupational burnout was not identified.

## Discussion

To our knowledge, this is the first study to investigate the neural correlates of perceived occupational stress using NIRS. Our study demonstrated that occupational burnout is associated with reduced cortical activity over the bilateral frontopolar cortex and DLPFC during a VFT. Occupational burnout was significantly associated with job strain and BDI score. Job control was not correlated with cortical activity. On the other hand, higher job demand was significantly correlated with reduced cortical activity at the left fronto-polar region. In addition, job strain was not associated with cortical activity over the bilateral frontotemporal regions but was instead significantly associated with occupational burnout and BDI scores. These findings support our hypothesis that occupational burnout was associated with reduced PFC activity during the VFT.

### Occupational burnout and PFC cortical activity

Similar to the previous NIRS “study”, we did not find any associations between job strain and cortical activity over the bilateral frontotemporal regions^10^. The JDC model defines job strain as the ratio between job demand and job control. It has been hypothesized that job demand and job control could exert different influences on the functional brain network, and therefore, the variable job strain may not reflect the real relationship between job-related stress and altered brain functions^10^. Future studies are warranted to confirm this hypothesis. On the other hand, we found that occupational burnout was correlated with reduced cortical activity over the bilateral frontopolar cortex and DLPFC. In previous MRI studies, some of these regions have decreased brain volume or function in individuals with occupational burnout, such as reduction in the GM volumes of the DLPFC^12^, decreased functional connectivity between the amygdala and DLPFC during an emotional regulation test^15^, and decreased BOLD signals in the right DLPFC during a clinical reasoning task^17^. The DLPFC is involved in various cognitive functions, including the monitoring and managing remembered information, implementation strategies to assist with memory, and working memory^21^,^22^,^23^. The DLPFC is responsible for the planning and sequence of behaviors required for goal-directed behavior. This process is crucial to the broad realm of cognitive control, and requires both the learning and implementing rules of behavior that lead to success, including modifying those rules if necessary^24^.

Our results also demonstrated that occupational burnout was associated with reduced cortical activity over the bilateral frontopolar cortex (FPC, BA10, or anterior FPC) during a VFT, which has not been previously reported. Brain imaging has provided evidence that FPC contributes to reasoning or problem-solving^25^,^26^ as well as learning, exploration, memory retrieval, relational reasoning, and multiple task coordination^27^. It has been hypothesized that the typical mode of behavior in subjects with occupational burnout consist of an underestimation of the significance of work load, acceptance of high workload, and a feeling that the work situation can be controlled by increasing the working hours resulting from maladaptive coping effort and strategy^3^. Therefore, it is reasonable to postulate that occupational burnout was associated with impaired DLPFC and FPC-mediated cognitive responses involving the appraisal and the coping of stress. Such changes could constitute a stress vulnerability factor, or be a consequence of prolonged occupational stress. We did not evaluate the causal relationship in our study, and future studies are warranted.

### Verbal fluency test and the prefrontal cortex

There are two versions of VFT based on the type of cue provided: the category fluency task (CFT) requires individuals to generate words belonging to a specific semantic category, while the letter fluency task (LFT) requires the generation of words based on phonemic cues^28^. In the present study, we demonstrated significantly increased cortical activities over bilateral frontotemporal regions during the LFT, a finding that agrees with that of previously reported studies using NIRS^29^,^30^,^31^. Furthermore, we identified significantly decreased cortical activity over the PFC during the LFT to be associated with occupational burnout.

Previous study using NIRS have demonstrated a differential pattern of modulation of brain activity for phonemic and semantic word generation, with equally increased cortical activities in the frontotemporal cortices for both types of VFT compared to a selective activation of the anterior and superior prefrontal areas with the LFT^28^. Therefore, future studies adopting CFT are warranted to elucidate the associations between occupational burnout and brain activity during semantic processing.

### Occupational burnout and C-JCQ parameters

Although we identified a significant correlation between occupational burnout and job strain, the correlation was weak (*r* = 0.374, *P* = 0.032). There are several possible explanations for this finding. First, we used the proxy measure of occupational burnout, which provides individual perceived rating of occupational stress. This subjective evaluation of stress when facing a stressful event (e.g., job strain) reflects a person’s appraisal of the situation, adequacy of his/her coping strategies, and his/her perceived level of control. Therefore, people may experience similar negative life events with different psychological reactions. Second, job strain is quantified as the ratio of job demand to job control. However, as previously mentioned, it is likely that job demand and job control could contribute differently to perceived occupational stress^32^, as well as influence different functional brain networks^10^. Our findings demonstrated that occupational burnout was significantly associated with job demand (*r* = 0.515, *P* = 0.000) but not with job control (*r* = 0.034, *P* = 0.782), which is consistent with this hypothesis.

### Sex differences in the PFC activity during a VFT

In addition, we found male was associated with increased cortical activity over the PFC, which was consistent with previous NIRS studies^33^,^34^,^35^. It was proposed that this finding was due to the difference in difficulty performing the same task between male and female subjects^36^; in general, female subjects perform language tasks more efficiently than male subjects^37^. Therefore, male subjects may require greater cognitive demands during VFT performance.

### Depression and the PFC cortical activity

The relationship between depression severity and PFC cortical activity during a VFT remains inconclusive. Some study demonstrated positive correlations between depression severity and the PFC cortical activity^38^, while others did not show significant associations^39^,^40^. In our study, depression severity was negatively associated with cortical activity of the PFC, which was consistent with findings reported by Sato and his colleagues^20^. These discrepancies were probably due to differences in the study subjects recruited, assessments tools, and methodologies used in these studies. However, one important question worth discussing is whether these results are confounded by the presence of depression. To address this, board-certified psychiatrists interviewed all participants using the Mini-International Neuropsychiatric Interview (MINI) to exclude psychiatric disorders including dysthymic or major depressive disorder. In addition, we conducted multiple regression analysis adjusting for possible confounders including BDI scores, and the negative relationship between occupational burnout and cortical activity remained significant. Moreover, due to the involvement of limbic networks in major depression and occupational burnout, emotional reactions to both conditions may share similar symptomology but represent different constructs^3^,^15^,^41^. Given that depression can be triggered by stress, it is also possible that these individuals were in the process of developing depression, and the level of which was subclinical at the time of evaluation^3^.

## Limitations

Our results should be viewed in light of several limitations. First, given the relatively small number of study participants, we may not have sufficient statistical power to detect differences between the sexes; studies with larger samples and more detailed observations are likely needed. Second, unlike Kawasaki *et al*.’s study that used a population-based social epidemiological survey, our study participants were recruited from a medical center and selection bias is possible^10^. Third, because we employed a cross-sectional design, we could not examine the longitudinal causal relationships between occupational burnout and cortical activity. Fourth, we cannot totally exclude the influences of physiological noises on NIRS signals as we did not use signal processing approaches such as common average reference, adaptive filtering or transfer function models^42^. Fifth, since most of the study participants were female, we did not investigate the effects of menstrual cycle on brain function, as it has been shown that estrogen and progesterone have a modulatory effect on brain activity^43^. Sixth, due to limited numbers of study subjects, we did not directly compare the brain activity between subjects in high and low stress group. Future studies recruiting more participants and categorizing subject groups based on cutoff value of Chinese version of the Copenhagen Burnout Inventory (C-CBI) scores would enable to reveal the differences of cortical activity between high and low stress groups. Finally, we did not investigate the effect of sleep duration on brain activity, as it has been shown that sleep duration previous night is positively correlated with brain activity^44^.

## Conclusion

In this study, we investigated the relationship between frontotemporal cortical activity and perceived occupational stress. We found an association between occupational burnout and reduced cortical activity over the bilateral FPC and DLPFC during a VFT. As such, this research demonstrated a possible neural basis of perceived occupational stress that are distributed across cortical areas of the PFC. Studies with longitudinal design, larger study sample, and wider breadth of behavioral assessments are required to confirm and extend our findings.

## Materials and Methods

### Study participants

A total of 68 participants (17 men, 51 women, age range 20–62 years) were recruited in this study (Table 2). All of them were right-handed, which was defined as >70 points in the Edinburgh Inventory^45^. All participants are native Chinese speakers and were recruited from various routes, including acquaintance of the authors, hospital staff and via other participants. Exclusion criteria were neurological illness, traumatic brain injury with any known cognitive consequence or loss of consciousness for more than 5 minutes, a history of mental retardation or alcohol/substance abuse/addiction, or history of a psychiatric disorder. To rule out any psychiatric disorders, two board-certified psychiatrists (P.H.C and W.H.L) interviewed all participants using the MINI^46^. This study complied with the Declaration of Helsinki, and was approved by the Institutional Review Board of Taichung Veterans General Hospital (approval No. CF14045). All participants received a complete explanation of the study and provided written informed consent. Information on age, gender, educational level, working status (full-time or part-time), socioeconomic status (SES) and the average number of working hours per day for the week prior to the NIRS measurement was obtained using a standardized questionnaire. In Taiwan, a minimum of a 35-hour workweek is used as the definition for full-time employment^47^. SES was assessed using the Hollingshead scale^48^.

### Clinical measurements

#### Beck Depression Inventory-II

Depression was assessed using the Chinese version of Beck Depression Inventory II (BDI-II)^49^. The BDI-II contains 21 questions assessing the severity of common depressive symptoms. For each item, participants are required to select an item on the point scale (from 0 to 3) that best describes how they felt in the last 2 weeks. Total scores on this measure ranged between 0 and 63, with higher scores indicating higher levels of depression.

#### Assessments of perceived occupational stress

In the present study, perceived occupational stress was evaluated using the C-CBI^50^ and C-JCQ.

##### Copenhagen Burnout Inventory

We used two parameters in the C-CBI^50^. The first was personal burnout, which measures the degree of physical and psychological exhaustion experienced by the person, regardless of occupational status. The second, occupational burnout, measures the degree of physical and psychological exhaustion perceived as related to work by the person. Detailed information regarding the English and Chinese versions of the CBI, including the details of all subscales, items, calculation formula, and validity can be found elsewhere^51^,^52^,^53^,^54^. In brief, the personal burnout scale contained five items: “How often do you feel tired?”, “How often are you physically exhausted?”, “How often are you emotionally exhausted?”, “How often do you think: ‘I can’t take it anymore?’”, and “How often do you feel weak and susceptible to illness?”. The occupational burnout scale contained 5 items: “Is your work emotionally exhausting?”, “Do you feel frustrated with work?”, “After a day of work, do you feel exhausted?”, “Do you feel tired just thinking about starting another day of work?”, and “Do you feel that every moment at work is hard?”. The responses were coded using a 5-point scale of “always”(score 100), “often” (75), “at times”(50), “not often”(25) and “never”(0).

##### Chinese version of Job Content Questionnaire

The C-JCQ included nine items for the job control scale (learning new things, non-repetitive work, creative work, allowing own decision, high level of skills, freedom to make decision, various tasks, influential opinions, and develop one’s abilities) and seven items for psychological job demands scale (fast work, hard work, excessive work, insufficient time, concentrate on job for long time, hectic work and insufficient manpower). Each item was listed as a statement, and the response was recorded on a four-point Likert scale, ranging from 1 (strongly disagree) to 4 (strongly agree). These two scales are based on Karasek’s Job Demand-Control (JDC) model^55^ and their Chinese version have been validated and widely used in Taiwan^56^,^57^,^58^,^59^. The JDC model postulates that increased job demands combined with decreased job control contributes to a strain that increases risk of stress-related illness^56^. Job strain was defined as the ratio between job demand and job control^9^.

### Cognitive activation test

We used a 160-s block-design VFT (letter version). VFT assesses the functions of the prefrontal cortex such as working memory, inhibition of inappropriate responding, the retrieval of items from long-term memory, and self-initiation^31^. It has been widely used in previous NIRS studies^33^,^34^,^35^,^60^. The detailed procedure during the VFT in the present study can be found in depth elsewhere^29^. In brief, the 160-s block-design contains three different time periods: a 30-s pre-task period, a 60-s task period, and a 70-s post-task period. The 60-s task period was comprised of three contiguous 20-s segments, that were initiated by a single syllable selected from nine possible options (first, /ㄅ(b)/, /ㄆ(p)/, or /ㄉ(d)/; second, /ㄊ(t)/, /ㄌ(l)/, or /ㄋ(n)/; third, /ㄇ(m)/, /ㄈ(f)/, or /ㄘ(dz)/). Individuals were instructed to say as many words as possible that started with a phonological syllable presented audibly from the computer and the total number of correct words generated during the task was recorded as an index of VFT performance.

### NIRS instrument

**Figure 3:**
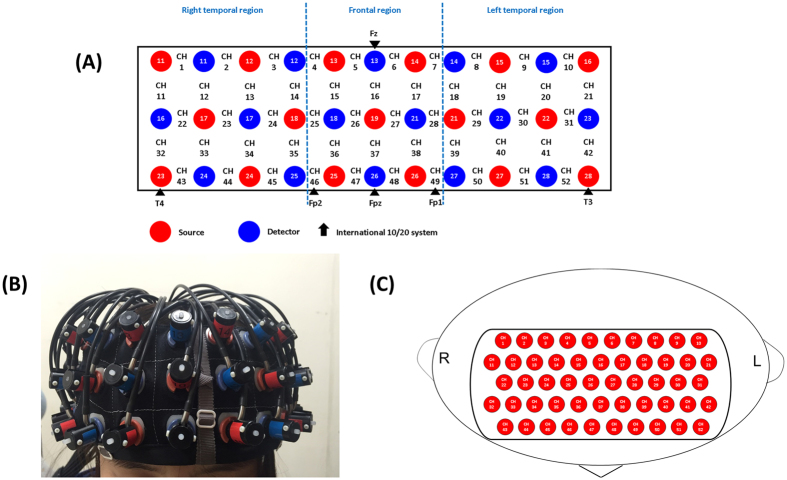
Probe setting and measurement points for 52-channel NIRS. (**A**) The localizations of all 52 channels were positioned according to the international 10–20 system. Red and blue circles indicate near-infrared light emitter and detector positions, respectively. By using the international 10–20 system, the detector 13 was positioned on the F z marker point, while the bottom row of channels was placed on a line between T 3 and T 4. (**B**) Probes with thermoplastic 3 × 11 shells were placed over bilateral frontotemporal regions. (**C**) The 52 measuring areas are labelled as ch1 to ch52 from the right posterior to left anterior.

We used a 52-channel NIRS instrument (ETG-4000; Hitachi Medical Co) to measure changes in hemoglobin concentration. The NIRS probe attachments were thermoplastic 3 × 11 shell sets with 52 channels (Fig. 3). The lowest probe line was set along the Fp1-Fp2 line, as defined by the international 10–20 system used in electroencephalography. This probe arrangement can measure hemoglobin changes in the approximate surface regions bilaterally in the DLPFC (Brodmann’s area [BA] 9 and 46), VLPFC (BA 44, 45 and 47), fronto-polar (BA 10), and anterior part of the temporal cortex (aTC; BA 21 and 22). The distance between pairs of source and detector probes was 3.0 cm. We defined the measurement area between each probe-set pair as one “channel”, which was sufficient to measure at depths between 20 and 30 mm from the scalp. This depth corresponds to the surface of the cerebral cortex. The NIRS instrument measures changes in both [oxy-Hb] and [deoxy-Hb] by using two wavelengths (695 and 830 nm) of near-infrared light (indicated as mM) on the basis of the Beer–Lambert law^61^.

The data sampling rate was 0.1 s using the integral mode. The pre-task baseline was determined as the mean over a 10-s period immediately before the task period, and the post-task baseline was determined as the mean over the last 5 s of the post-task period. Linear fitting was applied to the data between these two baselines. A moving average method using a 5-s window was applied, and artifacts were removed using an automatic artifact-rejection program in the NIRS instrument^31^. Because we excluded the rejected channels from further analysis, the number of available channels varied among individuals (the range of the number of rejected channels: 0–24, mean, 5.7; standard deviation, 6.1). The spatial information for each channel was estimated by using data from the Functional Brain Science Laboratory at Jichi Medical University in Japan^62^,^63^. In the present study, we used the mean changes in [oxy-Hb] measured during the VFT as an index of cortical activity. We chose [oxy-Hb] as an indicator because it better reflects cortical activity and demonstrates stronger correlations with fMRI blood-oxygenation level-dependent signals compared to [deoxy-Hb]^20^.

### Data analysis

First, to confirm the significant activation in 52 channels during the VFT, the mean [oxy-Hb] and [deoxy-Hb] changes from the pre-task baseline to the task period were compared using a paired Student’s t-test. As 52 paired t-tests were performed, the false discovery rate (FDR) method was used to correct for multiple comparisons (two-tailed; we set the value of q specifying the maximum FDR to 0.05, so that there are no more than 5% false positives on average)^64^. Second, we examined the relationship between occupational burnout scores and the mean [oxy-Hb] and [deoxy-Hb] changes measured in each channel during the cognitive task by using Pearson’s correlation coefficient corrected with the FDR. Since occupational burnout score is on an ordinal scale, we preliminarily examined its distribution for normality using the Shapiro-Wilk test (*P* > 0.05). Third, to elucidate the independent contributions of occupational burnout to mean [oxy-Hb] changes in the channels that had significant correlations, we performed stepwise multiple regression analyses. In these analyses, mean [oxy-Hb] change was the dependent variable. We controlled for other potential confounding variables such as gender (dummy parameterized, male = 1, female = 0), averaged working hours per day, age, VFT performance, personal burnout score, SES, JCQ parameters, and BDI scores, with a probability of F for a conservative entry and removal criteria of 0.05 and 0.1, respectively. For significant findings, effect sizes were indicated using the standardized regression coefficient (beta). Because multiple regressions focused on NIRS channels where brain activity was shown to be significantly correlated with occupational burnout after the FDR method, no further correction was applied and predictors were considered significant at *P* < 0.05^33^. Furthermore, we investigated the relationship between occupational burnout and personal burnout, SES, job strain, BDI scores, and VFT performance using Pearson’s correlation coefficient. In addition, we also examined the relationship between C-JCQ parameters and cortical activity. All statistical analyses were performed with SPSS 18.0 software (IBM Inc., Armonk, NY, USA).

## Additional Information

**How to cite this article**: Chou, P.-H. *et al*. Perceived Occupational Stress is associated with Decreased Cortical Activity of the Prefrontal Cortex: A Multichannel Near-Infrared Spectroscopy Study. *Sci. Rep.* **6**, 39089; doi: 10.1038/srep39089 (2016).

**Publisher's note:** Springer Nature remains neutral with regard to jurisdictional claims in published maps and institutional affiliations.

## Figures and Tables

**Table 1:** Summary of stepwise multiple regression analysis in channels showing significantly correlated with occupational burnout.

			Independent variables		
			Occupational burnout		Other variables
Channel No.	R^2^	Adjusted R^2^	beta	*P*	
Ch05	0.185	0.171			BDI: beta = −0.430, *P* < 0.001
Ch15	0.209	0.196			BDI: beta = −0.457, *P* < 0.001
Ch16	0.170	0.156			Job demand: beta = −0.412, *P* < 0.001
Ch26	0.265	0.241	−0.426	<0.001	Male: beta = 0.225, *P* = 0.044
Ch27	0.209	0.183	−0.316	0.008	Male: beta = 0.282, *P* = 0.017
Ch28	0.265	0.241			Male: beta = 0.356, *P* = 0.002; Personal burnout: beta = −0.318, *P* = 0.006
Ch36	0.145	0.131	−0.381	0.002	
Ch37	0.179	0.152	−0.311	0.010	Gender: beta = 0.250, *P* = 0.038
Ch38	0.147	0.132	−0.383	0.002	

No., Number; Ch, Channels; BDI, Beck Depression Inventory.

**Table 2:** Demographic and characteristics of study participants.

	N = 68	
	Mean	SD
Age	30.5	11.8
Gender (Male/Female)	(17/51)	
Education (Graduate/Under graduate/high school or below)	(4/56/8)	
LFT	12.8	5.3
Socioeconomic status	2.8	0.7
Average working hour per day	8.9	1.4
Working status (full time/part time)	(62/6)	
Occupational burnout score	41.8	16.8
Personal burnout score	43.8	19.4
Job demand	59.2	15.8
Job control	55.3	11.9
Job strain	2.2	0.8
BDI score	8.8	7.6

LFT: letter version of verbal fluency test; BDI: Beck depression inventory.
